# Genomic Profiling of Circulating Tumor DNA from Patients with Extensive-Stage Small Cell Lung Cancer Identifies Potentially Actionable Alterations

**DOI:** 10.7150/jca.55134

**Published:** 2021-06-22

**Authors:** Jing Yang, Xiangyun Wang, Jingli Lu, Hui Chen, Xiaochen Zhao, Chan Gao, Yuezong Bai, Qiwen Zhang, Xiaomin Fu, Xiaojian Zhang

**Affiliations:** 1 Department of Pharmacy, The First Affiliated Hospital of Zhengzhou University, Zhengzhou, China.; 2 Henan Key Laboratory of Precision Clinical Pharmacy, Zhengzhou University, Zhengzhou, China.; 3 Department of Respiratory and Critical Care Medicine, Second Affiliated Hospital of Naval Medical University, Shanghai.; 4 The Medical Department, 3D Medicines Inc., Shanghai, China.; 5 Department of Cancer Immunotherapy, The Affiliated Cancer Hospital of Zhengzhou University and Henan Cancer Hospital, Zhengzhou, China.

**Keywords:** ctDNA, small cell lung cancer (SCLC), genomic profiling, targetable alterations

## Abstract

Comprehensive genomic profiling may help uncover potentially actionable alterations in small cell lung cancer (SCLC) patients who have progressed on standard chemotherapy. However, tissue procurement may be extremely challenging for extensive-stage patients. We aimed to investigate the possibility of genomic profiling and detecting actionable alterations from blood in Chinese SCLC patients. Blood samples collected from extensive-stage SCLC pateints were subjected to circulating tumor DNA (ctDNA) extraction and targeted-next generation sequencing (NGS) using a 150-gene panel. A total of 1,300 aberrations were detected in 128 genes and 89.2% (116/130) patients harbored at least one oncogenic alteration. The most frequently mutated genes included *TP53* (82.3%), *RB1* (56.2%), *LRP1B* (40.8%) etc. and 54.6% of the patients had concurrent *TP53/RB1* mutations. The RTK/RAS/RAF axis was the most frequently mutated oncogenic pathway. Samples harboring alterations in the DNA damaging repair (DDR), Notch, PI3K/mTOR, RTK/RAS/RAF, and Wnt pathways exhibited significantly higher blood tumor mutational burden (bTMB) than their wildtype counterparts. Classification based on OncoKB criteria detected potentially actionable alterations in about 50% of the population, half of which were bTMB-H and bMSI-H, indicating response to immune checkpoint inhibitors. Alterations in the RTK/RAS/RAF, DDR, and PI3K/mTOR also suggested potential sensitivity to matched targeted therapies or emerging investigational agents. Blood-based panel NGS is promising for delineating genomic landscape of SCLC and may also shed some light on treatment selection for Chinese SCLC patients.

## Introduction

Small cell lung cancer (SCLC) is a highly malignant neuroendocrine tumor that accounts for up to 15% of all lung cancers 1. Although the majority of SCLC patients respond well to chemotherapy initially, nearly all patients develop resistance within months and their 5-year survival is only ~6% 2. In recent years, the advent of immune checkpoint inhibitors (ICIs) has greatly expanded the therapeutic armamentarium of SCLC. In recurrent SCLC patients treated with nivolumab ± ipilimumab or pembrolizumab, the efficacy was demonstrated to be highly correlated with tumor mutational burden levels 3, 4. In addition to immunotherapy, a number of targeted therapies are also being developed to treat SCLC, and there already exists some evidence showing off-label use of targeted agents benefiting SCLC patients 5. For instance, human epidermal growth factor receptor 2 (HER2)-positive relapsed patients and those carrying deleterious mutations in *BRCA1* responded favorably to trastuzumab- and olaparib-based regimens, respectively 6, 7. Therefore, next-generation sequencing (NGS)-based comprehensive genotyping is becoming increasingly important for guiding SCLC management.

However, most SCLC patients are diagnosed with extensive-stage disease and tissue availability may pose significant challenges for those seeking genomic characterization. Circulating tumor DNA (ctDNA) sequencing, as a non-invasive alternative, has proven highly concordant with tissue-based sequencing and its utility has been widely explored for guiding treatment, evaluating efficacy, and predicting progression in multiple malignancies 8-11. Recently, ctDNA sequencing has been applied by multiple studies to unveil potentially targetable mutations in Caucasian SCLC patients, however, little is known about the genomic landscape of Chinese SCLC population in blood 12-15. In this work, we profiled genomic alterations among 130 Chinese extensive-stage SCLC patients and more importantly, uncovered potentially actionable targets, the value of which to inform treatment decision warrant further validation in larger sample sizes.

## Materials and Methods

### Patients and sample collection

For genomic landscaping, patients were retrospectively included between January, 2015 and May, 2020 if they were histopathologically or cytologically diagnosed with extensive-stage SCLC and had sufficient peripheral blood for NGS analysis of ctDNA using a 150-gene panel (3D Medicines). Patients were excluded if they had other prior or concomitant tumors. For clinical validation of the potentially targetable alterations, an extensive-stage SCLC patient admitted at Changzheng Hospital, Shanghai was given matched targeted therapy according to NGS analysis of ctDNA. Ten milliliters of peripheral blood were collected from each patient for NGS testing. This study was carried out in accordance with the Declaration of Helsinki and informed consents were obtained from all participants.

### Circulating tumor DNA sequencing and variant calling

Circulating tumor DNA sequencing was performed using a panel targeting the whole coding regions of 150 cancer-related genes with a median sequencing depth of 3852 at 3D Medicines Inc., a College of American Pathologists (CAP) accredited and Clinical Laboratory Improvement Amendments (CLIA) certified laboratory (Shanghai, China). DNA isolation, library preparation, targeted hybrid capture and sequencing were performed as previously described 16. Libraries were prepared with 30-60 ng of cell-free DNA using the Accel-NGS 2S Plus DNA Library Kit (Swift Biosciences) and resulting DNA fragments were tagged with unique molecular identifiers (UMIs) to reduce background noise. Hybrid capture was conducted using the xGen Exome Research Panel v2 (Integrated DNA Technologies), followed by 75bp paired-end sequencing on a NextSeq 500 platform (Illumina).

Sequencing reads were mapped to the reference human genome hg19 using the Burrows-Wheller Aligner (v0.7.12). Duplicate reads were removed using Picard (v1.130). Synonymous mutations were excluded from analyses. Non-synonymous single nucleotide variants (SNVs) and insertions/deletions (indels) were called if their allele frequencies (AFs) were ≥0.3% using MuTect (v1.1.7) and Pindel (v0.2.5a8), respectively. Copy number variations (CNVs) were reported using an in-house developed script with a cut-off of 6 copies. Fusions were called using an in-house developed script with at least 3 supporting reads.

### Blood-TMB and blood-MSI determination and identification of actionable targets

Blood TMB (bTMB) was defined as the total number of somatic SNVs and indels in the coding regions examined, including missense, truncating and inframe mutations. Samples in the top quartile of bTMB levels (≥6) were classified as bTMB-H. Blood MSI (bMSI) status was also examined for 100 samples, where a panel of 100 microsatellite markers were evaluated using a proprietary algorithm and any sample with ≥ 20% unstable loci was defined as bMSI-H 17. Clinically actionable alterations were identified using the OncoKB Precision Oncology Database 18.

### Statistical analyses

Between-group differences in bTMB were assessed using a Mann-Whitney U test. The correlation in SNVs/indels frequency between our study and the NATURE_2015 cohort was examined using Pearson correlation test 19.

## Results

### Patient characteristics and the genomic landscape of Chinese SCLC patients in blood

**Figure 1:**
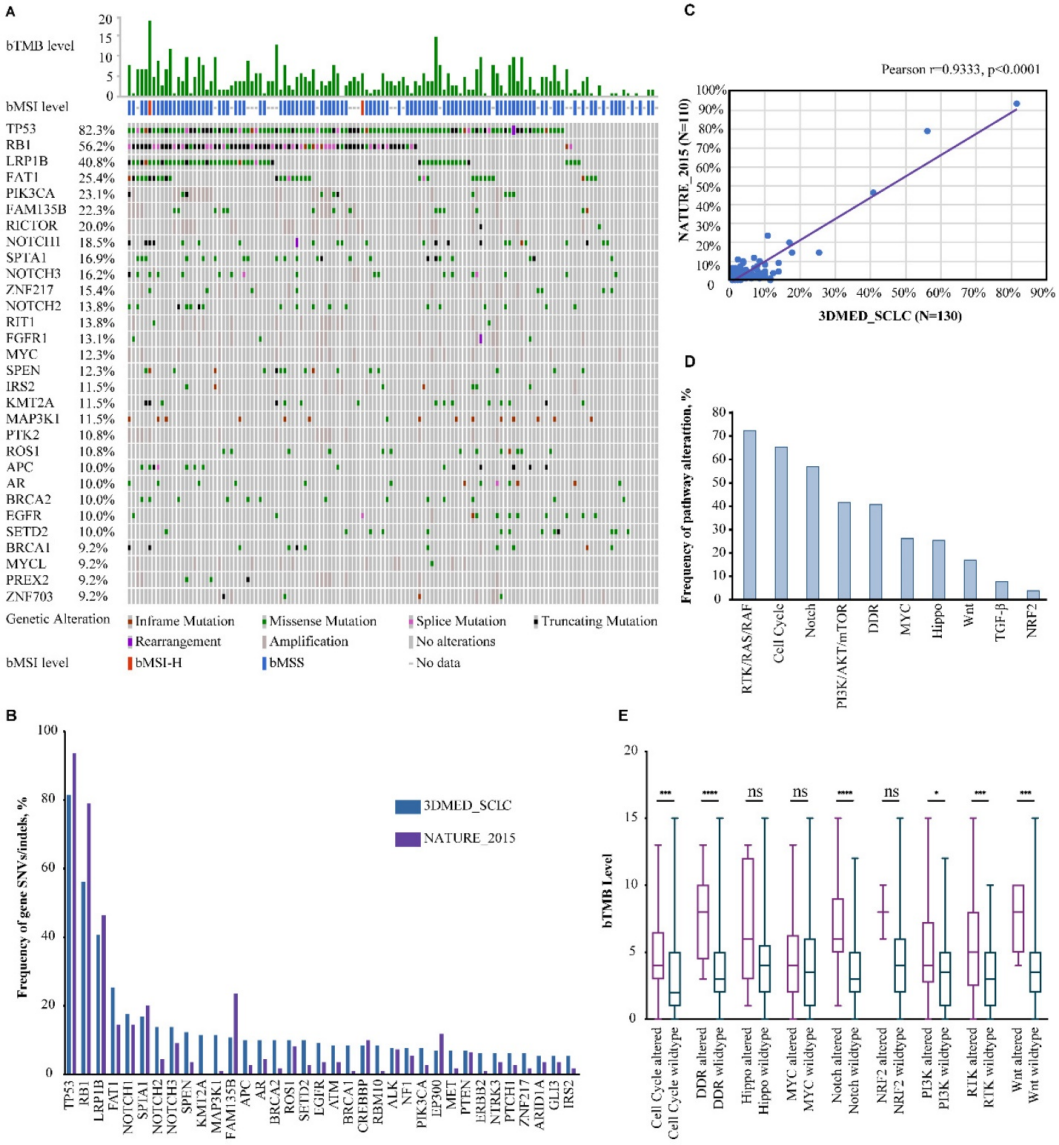
Significantly altered genes (**A**) and pathways (**D**) of 130 Chinese extensive-stage SCLC patients. **B.** The top 30 SNVs/indels in our cohort and the NATURE_2015 cohort. **C.** The correlation in mutation frequency between our cohort and the NATURE_2015 cohort. **E.** Correlation between bTMB level and mutated pathways. ns, not significant; *, p<0.05; ***, p<0.001; ****, p<0.0001.

A total of 130 blood samples were obtained from extensive-stage SCLC patients. The median age of the patients was 65 years (range 42-91) and 87.7% were male (**Table 1**). The median number of oncogenic alterations was 3 (range 0-15). Two of the 100 samples subjected to bMSI testing were bMSI-H and 36/130 (27.7%) samples were defined as bTMB-H. Overall, 1,300 aberrations were detected in 128 genes and 116 (89.2%) patients harbored at least one oncogenic alteration (**Fig. 1A**).

The most frequently mutated genes were *TP53* (82.3%), *RB1* (56.2%), *LRP1B* (40.8%), *FAT1* (25.4%), *PIK3CA* (23.1%), *FAM135B* (22.3%), *RICTOR* (20.0%), *NOTCH1* (18.5%), *SPTA1* (16.9%), and* NOTCH3* (16.2%). The most frequently amplified genes were *PIK3CA* in chromosome (chr) 3 (19.2%), *RICTOR* in chr 5 (18.5%), *MYC* in chr 8 (12.3%), *RIT1* in chr 1 (12.3%), and *FAM135B* in chr 8 (11.5%). Gene rearrangement was detected in 5 samples: *NOTCH1-LCNL1* (chr 9), *PTEN*-intragenic (chr 10), *TNRC6B-EP300* (chr 22), *TP53* (chr 17)-*DHRS3* (chr 1), and *FGFR1-PLEKHA2* (chr 8), of which *PTEN*-intragenic is likely to cause loss of function while others were all likely-oncogenic. The mutational landscape was highly consistent with that of an external SCLC cohort, NATURE_2015 (Pearson r=0.9333, p<0.0001) (**Figure 1B&C**) 19.

As inactivating mutations of tumor suppressor genes *TP53* and *RB1* were both reported to be obligatory in SCLC, we analyzed the co-mutation rate of these two genes 19. Of the 130 samples, 71 (54.6%) had concurrent *TP53/RB1* alterations while 36 and 2 were mutated only in *TP53* and *RB1*, respectively. SNVs/indels in *LRP1B, SPEN* and *ATM* and amplifications of *PIK3CA, FAM135B, RICTOR, RIT1, PTK2, MYCL, DDR2, NTRK1, CCNE1* and *CD274* were more common among *TP53/RB1* co-mutated samples.

### Alterations in cancer-related signaling pathways and their correlations with bTMB

To gain more insight into the molecular mechanism underlying SCLC pathogenesis, the alterational profiles of 10 critical oncogenic signaling pathways were also investigated 20, 21. Strikingly, 72.3% of the patients carried at least one alteration in the RTK/RAS/RAF pathway (**Fig. 1D**). SNVs/indels in *ROS1, EGFR, ALK, NF1,* and *MET* occurred in 10.0%, 9.2%, 7.7%, 7.7%, and 6.9% of the study population and amplifications of *RIT1, FGFR1, IRS2, NTRK1,* and *BRAF* were observed in 12.3%, 10.0%, 7.7%, 5.4%, and 3.1% of the patients (**Table 2**). Similar to previous reports, alterations in the cell cycle (65.4%), Notch (56.9%), PI3K/mTOR (41.5%), DNA damaging repair (DDR) (40.8%) and MYC (26.2%) pathways were also among the most mutated pathways 13, 22. DDR alterations have been reported to be associated with higher TMB loads in several cancers, therefore, the correlation between alterations in DDR as well as other signaling pathways and TMB status was also explored. With bMSI-H samples excluded, samples carrying alterations in the cell cycle, DDR, Notch, PI3K/mTOR, RTK/RAS/RAF, and Wnt pathways had significantly higher bTMB than their wild-type counterparts (**Fig. 1E**).

### Potentially actionable genomic alterations

**Figure 2:**
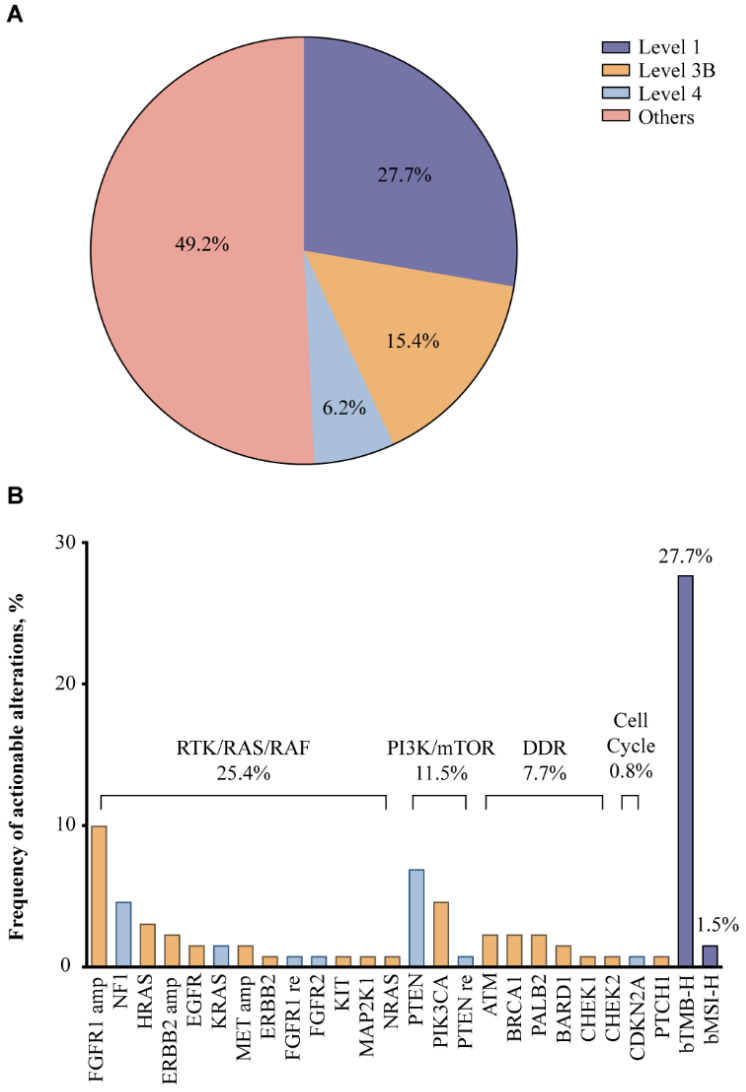
Potentially actionable alterations based on OncoKB criteria (A) and the frequency of potentially actionable alterations (B) in 130 Chinese extensive-stage SCLC patients.

According to OncoKB classification, potentially actionable alterations were detected in about 50% of the patients (**Fig. 2A**). Level 1 markers included bTMB-H and bMSI-H, accounting for 27.7% of the study population, indicating that these patients may benefit from ICIs (**Fig. 2B**). Around 15.4% of the patients harbored level 3B alterations such as SNVs/indels of *PIK3CA*, *HRAS*, *ATM, BRCA1* and *PALB2*, and amplifications of *FGFR1, HER2,* and* MET*, while level 4 alterations were found in 6.2% of the patients, including SNVs/indels of *PTEN, NF1* and* KRAS*. Most of these potentially targetable genes could be categorized into oncogenic pathways: RTK/RAS/RAF pathway (25.4%), PI3K/mTOR pathway (11.5%), and DDR pathways, more specifically the homologous recombination (HR) pathway (7.7%). Given previous success with therapeutic agents targeting the RTK/RAS/RAF and PI3K/mTOR pathways in other malignancies and multiple emerging investigational agents in SCLC, alterations in these pathways may predict sensitivity to targeted therapies 23-25. In addition, DDR pathway alterations may also predict benefit from ICIs, considering accumulating evidence on improved efficacy of ICIs in DDR-mutated NSCLC 26.

**Figure 3:**
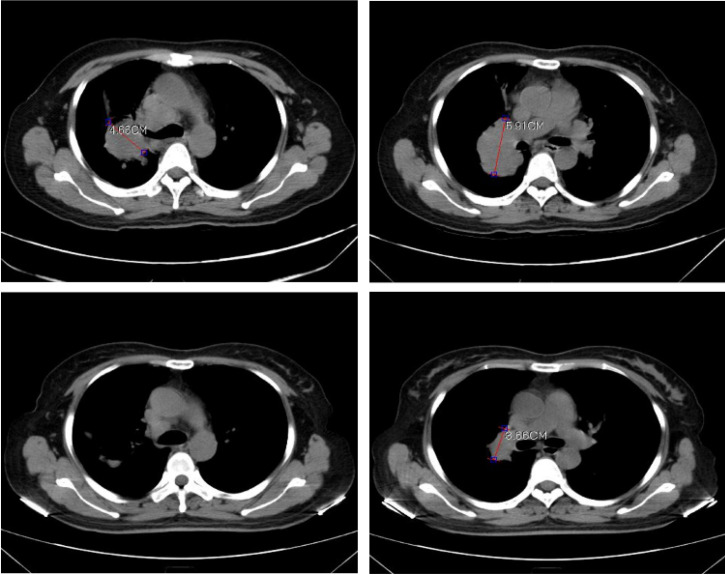
Computerized tomography (CT) scans showing tumor regression in the right upper lobe lesion and the right hilar lesion following treatment with osimeritinib in an extensive-stage SCLC patient harboring *EGFR* L858R and T790M mutations.

In order to validate the feasibility of leveargeing ctDNA-based testing to guide targeted therapy in SCLC, a 49-year-old female with a confirmed diagnosis of extensive-stage SCLC was subjected to NGS analysis of her peripheral blood using the 150-gene panel described above. The patient presented with a right upper lobe lesion measuring 4.66 cm, a right hilar lesion measuring 5.91 cm and multiple hepatic and bone metastases and had failed two cycles of chemotherapy with etoposide plus cisplatin previously. NGS anlaysis revealed mutations in *EGFR* (L858R at 56.4% and T790M at 55.14%), *ATM* (R3008H at 0.52%), *PTEN* (R130Q at 56.82% and S59* at 8.92%; * denotes a stop codon), and *TP53* (P278S at 68.89%). Considering the co-occurrence of L858R and T790M, the patient was started on osimertinib (80 mg/day, po) on June 1^st^, 2020. A computerized tomography (CT) scan conducted on June 26^th^, 2020 showed that the patient had achieved partial response (PR) with almost complete regression in the right upper lobe lesion and a 38.1% reduction in the right hilar lesion (**Figure 3**). Blood tests performed on July 2^nd^, 2020 also showed a remarkable drop in the levels of tumor biomarkers since the onset of osimertinib treatment (**Table 3**). The patient continued to receive osimertinib until disease progression (PD) as shown by a CT scan on September 22^nd^, 2020.

## Discussion

Our analyses revealed that genomic profiling using ctDNA may effectively capture the genomic complexity of SCLC by detecting 1,300 mutations in 128 genes. The most frequently altered genes included *TP53*, *RB1*, *LRP1B* etc., and over two thirds of the patients carried aberrations in the RTK/RAS/RAF pathway. Altertions in critical oncogenic pathways were also correlated with a higher bTMB load. More importantly, around 50% of the patients carried potentially actionable alterations, half of which might be sensitive to ICIs.

Blood-based genomic characterization of Chinese SCLC patients has been reported previously, but that study only included 22 patients and primarily focused on delineating clonal evolution 15. As for studies conducted in Caucasian populations, Carter L *et al* only included 31 patients, and copy-number aberrations in circulating tumor cells, not ctDNA, were used to differentiate chemosensitive from chemorefractory disease 27. Although Mohan S *et al* reported potential therapeutic targets in over 50% of the 69 patients included, only 30 had extensive-stage disease and ICI was not proposed as a treatment option 12. In contrast, with a cohort of 130, our study represents the largest study interrogating genomic features among Chinese SCLC patients from blood. We also discovered that around 30% (bTMB-H and DDR-mutants) of the patients may respond to ICI. It is true that the study by Devarakonda S. *et al* shared some similarities with our study, but they used four different panels (54-gene to 73-gene) for genomic profiling while our entire cohort was analyzed using a 150-gene panel, which was previously validated for bTMB determination in lung cancer 16. Therefore, bTMB-H was identified as an important predictor of ICI efficacy in our cohort.

Loss of *TP53* and* RB1*, often co-occurring, are almost universal in SCLC according to tissue sequencing 19. Indeed, *TP53* mutation was present in 76.3% of the cohort, but *RB1* was only mutated in 56.2%, however, when only SNVs/indels were taken into account, our result was only slightly lower than the 64% previously reported for Chinese SCLC patients in blood, although both were lower than the incidence of SNVs/indels based on tissue-testing 15, 22. This was not completely unprecedented as Devarakonda S. *et al* also observed a remarkably lower *RB1* mutation frequency from the blood of Caucasian SCLC patients compared to tissue testing 13. This inconsistency could have arised from population differences, a low fraction of ctDNA, challenges of detecting certain variations from ctDNA, etc.

The unusually high prevalence of RTK/RAS/RAF pathway alterations also caught our attention, while alterations in the cell cycle, Wnt and PI3K/mTOR signaling pathways were most common according to tissue analyses in Chinese SCLC patients 22. Again, Devarakonda S. *et al* had a similar finding in their ctDNA profiling, indicating the importance of RTK/RAS/RAF pathway in SCLC 13. This is consistent with previous literature showing the essential role of the RTK/RAS/MAPK signaling in tumor growth, metastasis, and metabolism of SCLC 28. Amplifications of* FGFR1, HER2,* and *MET* of this pathway as detected in our study may suggest therapeutic opportunities, given the emerging investigational agents or approved drugs targeting these aberrations either in SCLC or in other solid tumors 29, 30. Indeed, this notion was supported by a chemo-resistant SCLC case achieving PR following administration of osimertinib upon detection of T790M in *EGFR* from blood. Likewise, alterations in HR pathway genes such as *BRCA1, ATM, PALB2,* etc. may suggest sensitivity to poly (ADP-ribose) polymerase (PARP) inhibitors olaparib and talazoparib, both of which are being investigated for treating HR pathway-mutated (NCT03009682) or *BRCA1*-mutated SCLC 31. Apart from matched targeted therapies, alterations in the Notch and DDR pathways may also predict benefit from ICIs, as they were both found to be associated with higher bTMB. Notch pathway alterations were reported to be correlated with a higher degree of tumor immune infiltration and improved response to ICI in non-small cell lung cancer 32. We also for the first time reported two cases of bMSI-H SCLC, both of which were also classified as bTMB-H. Although rarely seen in SCLC, a bMSI-H phenotype may potentially predict response to ICI treatment.

Our study is limited by the retrospective design and the lack of paried tumor tissue. Patients' demographic data and treatment data were also missing, which prevented us from analyzing the correlation between genomic alterations and demographic characteristics/efficacy of matched treatment. Clonal hematopoiesis might have also interefered with alteration calling, which is a common issue with blood-based testing. Additionally, only one case received matched targeted therapy according to testing results, and the value of ctDNA analysis to guide clinical decision needs to be confirmed in larger studies. That being said, our work confirmed the feasibility of ctDNA testing in SCLC and provided us with more insight into pontetial targets for drug development.

## Figures and Tables

**Table 1:** Patient characteristics

Characteristics	
Patients	130
Median age, y (range)	65 (42-91)
**Sex**	
Female	16 (12.3%)
Male	114 (87.7%)
**bMSI status**	
bMSI-H	2 (1.5%)
bMSS	98 (75.4%)
N/A	30 (23.1%)
**bTMB status**	
bTMB-H	36 (27.7%)
bTMB-L	94 (72.3%)
Median oncogenic GAs (range)	3 (0-15)

**Table 2:** Alteration in RTK/RAS/RAF signaling

Gene	SNV/indel, N (%)	Amplification, N (%)
*ROS1*	13 (10%)	1 (0.8%)
*EGFR*	12 (9.2%)	2 (1.5%)
*ALK*	10 (7.7%)	0
*NF1*	10 (7.7%)	1 (0.8%)
*MET*	9 (6.9%)	2 (1.5%)
*HER2*	8 (6.2%)	3 (2.3%)
*NTRK3*	8 (6.2%)	1 (0.8%)
*IRS2*	7 (5.4%)	10 (7.7%)
*BRAF*	6 (4.6%)	4 (3.1%)
*ERBB4*	5 (3.8%)	0
*HRAS*	4 (3.1%)	0
*PDGFRA*	4 (3.1%)	0
*PDGFRB*	4 (3.1%)	0
*RAF1*	4 (3.1%)	0
*CBL*	3 (2.3%)	0
*FGFR1*	3 (2.3%)	13 (10%)
*FGFR2*	3 (2.3%)	1 (0.8%)
*FGFR3*	3 (2.3%)	0
*KRAS*	3 (2.3%)	2 (1.5%)
*NTRK1*	3 (2.3%)	7 (5.4%)
*NTRK2*	3 (2.3%)	0
*PTPN11*	3 (2.3%)	0
*JAK2*	2 (1.5%)	1 (0.8%)
*RET*	2 (1.5%)	3 (2.3%)
*RIT1*	2 (1.5%)	16 (12.3%)
*ARAF*	1 (0.8%)	0
*ERRFI1*	1 (0.8%)	3 (2.3%)
*KIT*	1 (0.8%)	0
*MAP2K1*	1 (0.8%)	1 (0.8%)
*NRAS*	1 (0.8%)	1 (0.8%)

**Table 3:** Levels of tumor biomarkers before and after osimertinib treatment

Biomarker	06-02-2020	07-02-2020	Reference range
NSE	166.6 μg/L	14.2 μg/L	< 16.3 μg/L
CYFRA211	122.6 μg/L	1. 7 μg/L	< 3.3 μg/L
CEA	1877 μg/L	90.09 μg/L	0-5 μg/L
CA19-9	2662 U/ml	111.90 U/ml	0-39 U/ml
CA125	562.2 U/ml	33.32 U/ml	0-35 U/ml
SCC	1.2 ng/ml	0.4 ng/ml	0-1.5 U/ml

## Abbreviations

SCLC: small cell lung cancer
NGS: next-generation sequencing
DDR: DNA damaging repair
ctDNA: circulating tumor DNA
ICI: immune checkpoint inhibitor
HER-2: human epidermal growth factor receptor 2
UMI: unique molecular identifier
indels: insertions and deletions
CNV: copy number variation
SNV: single nucleotide variation
AF: allele frequency
bTMB: blood tumor mutational burden
bMSI: blood microsatellite instability
HR: homologous recombination
